# HDL Cholesterol as a Marker of Disease Severity and Prognosis in Patients with Pulmonary Arterial Hypertension

**DOI:** 10.3390/ijms20143514

**Published:** 2019-07-18

**Authors:** Kamil Jonas, Grzegorz Kopeć

**Affiliations:** Department of Cardiac and Vascular Diseases, Jagiellonian University Medical College, John Paul II Hospital, 31-202 Krakow, Poland

**Keywords:** lipoprotein metabolism, inflammation, endothelium, microRNA

## Abstract

The impact of high-density lipoprotein (HDL) cholesterol on the development of atherosclerosis and diseases of systemic circulation has been well documented both in experimental and registry studies. Recent discoveries in pulmonary arterial hypertension (PAH) revealed a significant impact of HDL on pulmonary artery vasoreactivity and patients’ prognosis. The vasoprotective activity of HDL primarily involves vascular endothelium that also plays a central role in pulmonary arterial hypertension (PAH) pathobiology. However, the exact mechanism in which this lipoprotein fraction exerts its effect in pulmonary circulation is still under investigation. This paper reviews potential vasoprotective mechanisms of HDL in pulmonary circulation and presents current clinical reports on the role of HDL in PAH patients.

## 1. Protective Role of HDL in the Systemic Circulation

The significance of plasma high-density lipoprotein (HDL) cholesterol concentration in the development and progression of diseases has been documented by reports from both experimental and observational studies. The strong inverse relationship between HDL and cardiovascular disease was first established by the Framingham Heart Study [1]. This observation led to the hypothesis that HDL cholesterol might demonstrate protective properties against coronary artery disease. Further, it was supported by a series of animal studies showing inhibition of atherosclerosis in cholesterol-fed rabbits after HDL infusion [2] and in transgenic mice overexpressing apolipoprotein A-I (apoA-I) [3], which is the major protein component of HDL particles. A number of prospective studies in different populations have confirmed a strong and independent role of decreased HDL in the prediction of adverse cardiovascular events in the secondary prevention settings [4,5]. Despite the fact that low plasma levels of HDL are closely associated with increased risk of cardiovascular events, elevated HDL does not always provide clear protection against atherosclerosis. The challenges to the HDL hypothesis of cardiovascular diseases are also driven by studies in patients with Mendelian disorders of lipoprotein metabolism. Mutations in apoA-I, ATP-binding cassette transporter A1 and lecithin:cholesterol acyltransferase can lead to extremely low HDL levels but usually without advanced coronary artery disease [6,7,8,9]. Conversely, deficiency in cholesteryl ester transfer protein (CETP) activity resulting in increased HDL levels does not unequivocally protect against atherosclerosis [10]. Similarly, mutations resulting in structural alterations of apoA-I (e.g., Milano variant) are associated with decreased HDL concentration but not with coronary artery disease [11]. In addition to the abovementioned studies, several randomized studies aiming to improve HDL concentration failed to improve patients’ outcomes in the current statin era. Despite the previous reports suggesting benefits of increasing HDL concentration with nicotinic acid [12], current trials with extended-release niacin as an add-on therapy to the background statin failed to reduce the risk of cardiovascular events [13,14]. Previous trials with CETP inhibitors: Torcetrapib and dalcetrapib failed to show the beneficial effect of increasing HDL on cardiovascular risk [15,16]. Following studies investigating new CETP inhibitors showed equivocal results. While the use of anacetrapib decreased the risk of major cardiovascular events [17], treatment with evacetrapib did not provide similar benefits in high-risk patients [18]. The recent meta-analysis with the Mendelian randomization approach also showed that increased HDL associated with CETP gene polymorphism cannot be translated into reduction of cardiovascular risk [19]. What is more, in the dal-ACUTE study with dalcetrapib, authors have shown the dissociation between an increase of HDL levels and improvements in HDL function [20]. This highlighted the importance of HDL function in cardiovascular protection, which cannot be accurately estimated only by measuring lipoprotein concentration [21,22].

## 2. Pulmonary Arterial Hypertension

Pulmonary hypertension (PH) is defined as an increase in mean pulmonary arterial pressure ≥25 mmHg at rest as measured in right heart catheterization. Pulmonary arterial hypertension (PAH) describes a subject with pre-capillary PH (pulmonary artery wedge pressure ≤15 mmHg and a pulmonary vascular resistance >3 Wood units) in the absence of conditions such as PH due to lung diseases, chronic thromboembolic pulmonary hypertension, or other rare diseases [23]. PAH is a severe and progressive disease in which pulmonary vascular cells undergo uncontrolled hyperplasia leading to the narrowing and obliteration of small pulmonary arteries. Due to endothelial dysfunction, endogenous production of pulmonary vasodilators, including nitric oxide and prostanoids, is downregulated while increased levels of vasoconstrictors, including endothelin-1, contribute to vascular remodeling. This results in an increase of pulmonary vascular resistance, elevation of pulmonary artery pressure, and eventually heart failure [24,25]. PAH may be associated with different medical conditions, including HIV, connective tissue disease, heart defects, drug and stimulant intake and portal hypertension. In patients without any known predisposing factors or positive family history, idiopathic PAH (IPAH) can be diagnosed [23]. The first symptoms of PAH are mostly related to right ventricular dysfunction and are typically present during exertion. Most common symptoms of PAH include progressive shortness of breath, weakness, fatigue, angina, and syncope. The first line evaluation usually includes echocardiography, which can reveal the effects of PAH on the heart. This, however, always requires complementary non-invasive diagnostics and cardiac catheterization to establish the final diagnosis and support a treatment decision [23]. Currently available PAH-specific therapies are directed towards known pathomechanisms leading to pulmonary vascular disease and include calcium channel blockers, endothelin receptor antagonists, phosphodiesterase type 5 inhibitors, guanylate cyclase stimulators, prostacyclin analogues, and prostacyclin receptor agonists. While the contemporary survival of PAH patients is constantly improving due to the latest development of PAH-specific therapies, this condition is still considered as incurable [26]. To optimize patients’ care, a comprehensive assessment of treated patients is regularly performed in the reference centers. Ongoing clinical trials and large PAH registries including REVEAL [27], COMPERA [28], SPAHR [29], French registry [30], and many others enabled the formation and validation risk assessment tools incorporating multiple clinical (signs and symptoms of right heart failure, WHO functional class, six-minute walk distance, cardiopulmonary exercise testing), laboratory (N-terminal pro-brain natriuretic peptide concentration), imaging (right atrial area, presence of pericardial effusion in echocardiography, or cardiac magnetic resonance), and hemodynamic parameters (right atrial pressure, cardiac index, and mixed venous oxygen saturation), since there is no single variable that provides sufficient prognostic information. Based on the abovementioned parameters, the European PH guidelines formed a risk assessment tool widely used to stratify patients into low, intermediate, or high-risk groups according to estimated one-year mortality [23]. Classification and characteristics of different types of PH are presented in detail in Table 1.

## 3. HDL-Mediated Mechanisms of Cardiovascular Protection in Pulmonary Hypertension

Recently, PAH has been considered as a systemic disease due to the involvement of numerous organs and tissues and metabolic and inflammatory abnormalities [31,32,33]. Its etiology still remains unclear; however, recent studies revealed a significant role of endothelial dysfunction, inflammation, and altered metabolism in an onset and progression of the disease. Decreased HDL level and insulin resistance defined as increased triglyceride to HDL ratio was found to be more prevalent in the PAH population than in age-matched controls [34,35,36]. This was also associated with poor survival [34,37] and more advanced pulmonary vascular disease [38]. Vasoprotective properties of HDL primarily involve vascular endothelium that also plays a central role in PAH pathobiology. Traditionally, HDL capability to prevent atherosclerosis in the systemic circulation is explained by reverse cholesterol transport (RCT) allowing a transfer of cholesterol from the arterial wall to the liver [39,40]. A growing body of evidence indicates, however, a significant role of functions other than RCT vasoprotective functions of HDL that may play an important role in pulmonary circulation. Potential mechanisms of HDL-dependent vascular protection are summarized in Table 2.

## 4. HDL in NO and Prostacyclin Pathway

The molecular mechanism explaining the role of HDL in maintaining optimal pulmonary vascular resistance was identified as vasodilation via endothelial nitric oxide (NO) and prostacyclin release. Worth mentioning is the fact that most of the currently used PAH-specific medications, including phosphodiesterase 5 inhibitors, direct stimulators of soluble guanylate cyclase, and prostacyclin analogues, act on vascular endothelium by enhancing NO and prostacyclin signaling [41,42]. In the study by Nofer et al., HDL was shown to stimulate NO release from human endothelial cells and promote vasodilation in isolated aorta. These effects were ascribed to three lysophospholipids present in HDL: Sphingosylphosphorylcholine, sphingosine-1-phosphate, and lysosulfatide. All mentioned were able to elevate intracellular Ca2+ concentration, resulting in NO release and vascular relaxation. What is more, deficiency of the lysophospholipid receptor S1P3 decreased these vasodilatory effects of HDL by 60% [41]. Another study by Yuhanna et al. showed that endothelial NO synthase (eNOS) is stimulated by HDL in cultured endothelial cells but not by LDL cholesterol. Further expression experiments in animal models revealed that HDL signaling is mediated by scavenger receptor-BI (SR-BI), and subsequent phosphorylation of eNOS. HDL activation of eNOS was demonstrated in endothelial cell caveolae where both SR-BI and eNOS are localized. The addition of SR-BI and apo-AI antibodies was able to inhibit the response of isolated plasma membranes. HDL was also able to enhance nitric-oxide-dependent vascular relaxation in aorta of wild-type mice, but not in vessels from mice with homozygous null SR-BI knockout. This indicates that HDL can activate eNOS via SR-BI through a process that requires apoA-I binding [43]. This was further confirmed by extended study examining the molecular mechanism responsible for the increase in nitric oxide production. The HDL/SR-BI-dependent stimulation of eNOS was mediated mainly by an increase in intracellular ceramide levels independent of an increase in intracellular calcium or Akt kinase phosphorylation [44]. Another HDL-dependent pathway leading to vasodilatation is mediated by ATP-binding cassette G1 (ABCG1), which is a member of the large family of lipid, drug, and metabolite transporters. The ABCG1 role was initially associated with mediating cholesterol efflux from macrophages and maintaining tissue lipid homeostasis. Recently, the role of ABCG1 was established in modulation of different aspects of glucose and lipid metabolism. By controlling secretion and activity of lipoprotein lipase and insulin, ABCG1 can impact the risk of cardiovascular diseases, including pulmonary circulation. Worth mentioning is the fact that most of the data on vasodilatatory functions of HDL are derived from animal models of systemic circulation [45]. Previous studies on peripheral artery reactivity in subjects with intermediate and high cardiovascular risk confirmed a strong association between both HDL cholesterol and apo-AI concentrations, and vasoreactivity of small resistance arteries [46].

## 5. HDL and Inflammation

HDL also displays potent anti-inflammatory properties. It is worth noticing that, in animal models of PAH, both inflammation and altered immunity induce vascular remodeling. Importantly, it was shown that both pulmonary vascular cells and inflammatory cells can be an important source of inflammatory cytokines that lead to vascular contractility, proliferation, and remodeling typical for PAH. As recently shown, interleukin(IL)-1 can induce fibroblast growth factor 2 and, together with IL-6, plays a central role in promoting the proliferation of fibroblasts and smooth muscle cells of the pulmonary vasculature in PAH [47]. HDL particles and reconstituted HDL are effective inhibitors of cytokine-induced expression of vascular and intercellular adhesion molecules. The endothelial cell expression of these molecules has been further implicated in the recruitment of monocytes and the development of cardiovascular disease in in-vivo and in-vitro models [48]. T-lymphocytes may exert an inflammatory effect through significant up-regulation of IL-1β, tumor necrosis factor-α (TNF-α), and nuclear factor kappa-light-chain-enhancer of activated B cells signaling. This mechanism is likely to be involved in the pathogenesis of chronic inflammatory disorders leading to tissue destruction and development of pulmonary vascular disease. HDL by interacting with T-lymphocytes can inhibit monocyte stimulation and production of inflammatory cytokines. This anti-inflammatory function of HDL is attributed mainly to their main protein component, apoA–I, which binds activating factors at the surface of stimulated T cells [49]. HDL was also able to suppress interferon gamma production in human macrophages [50] and to induce macrophage transcriptional regulator ATF3, which resulted in toll-like receptor-dependent attenuation of inflammation [51].

This represents a potent mechanism in which HDL can modulate both innate and adaptive immunity and therefore prevent pulmonary vascular remodeling. What is more, HDL can bind circulating lipopolysaccharides (LPS), facilitate its hepatic clearance, and prevent LPS-induced endotoxemia. HDL also exhibits a potent impact on monocyte differentiation and IL-1β secretion in response to LPS in metabolic syndrome patients [52]. The vasoprotective and anti-inflammatory role of HDL was recently attributed to its bioactive components, including sphingosine 1-phosphate (S1P), which can regulate vascular functions and modulate immunity. S1P vascular signaling can be mediated by different G-protein-coupled receptors (S1P_1_–S1P_5_) resulting in contradictory effects on vascular remodeling. Some studies documented that S1P can induce vasoconstriction and development of pulmonary vascular disease via S1P_2_ receptors [53]. The other showed that stimulation of endothelial S1P_1_ receptor with HDL-bound S1P was able to mitigate inflammation in both in-vitro and in-vivo models by downregulation of adhesion molecules. Interestingly, albumin-bound S1P failed to exert a similar anti-inflammatory effect [54].

HDL-mediated anti-inflammatory properties are also closely associated with HDL’s ability to protect LDL and other lipoproteins from free radical-induced oxidative damage. Selective increase of HDL particles displaying decreased surface rigidity and rich in apoA-I were able to attenuate oxidative damage and mitigate inflammation. The antioxidant pathway includes a transfer of altered lipids from LDL to HDL particles and subsequent reduction of oxidized lipids by apoA-I and other HDL components [55]. These position the HDL particle as a major transport vehicle and redox-active component able to prevent vascular cell injury [56]. The antioxidant and anti-inflammatory role of HDL was recently confirmed also in subjects with PAH. Markers of oxidant stress may potentially contribute to the pathogenesis of PAH by inhibiting endothelial prostacyclin release, which plays a central role in pulmonary circulation. Ross et al. described proinflammatory HDL dysfunction not only in patients with connective tissue disease-associated PAH but also in the IPAH cohort. A significant increase in plasma levels of oxidation products of arachidonic and linoleic acid suggests an oxidant stress contribution to the proinflammatory HDL dysfunction in this disease. Interestingly, the HDL proinflammatory effects were mitigated by the treatment with an apoA-I mimetic peptide 4F, which may represent a novel potential PAH therapeutic target [57]. Worth mentioning is HDL’s ability to protect endothelial cells and macrophages from apoptosis. The preincubation of human endothelial cells with HDL before incubation with TNF-α was able to prevent apoptosis in a dose-dependent manner [58]. Other studies documented HDL-dependent cell protection from deleterious factors, including cholesterol loading, activation of complement system, and withdrawal of growth factor [40].

## 6. HDL and Insulin Resistance

In the last years, impaired glucose and insulin metabolism was proposed as a novel risk factor in pathobiology of PAH [34,37]. Recent studies in PAH patients reported a specific pattern of metabolic alterations, including insulin resistance defined as elevated triglyceride to HDL cholesterol ratio [34,59], glucose intolerance, and a blunted insulin response to an oral glucose load [60]. The exact link between altered glucose metabolism and smooth muscle cell proliferation in pulmonary vascular disease is still under investigation. A study comparing pulmonary artery smooth muscle cells (PASMCs) of IPAH patients and controls identified increased activation of the hexosamine biosynthetic pathway resulting in upregulated glycosylation and proliferation of PASMCs, leading to development of typical vascular hallmarks of PAH. What is more, a partial knockdown of this metabolic pathway in PASMCs of IPAH patients resulted in reduced PASMC proliferation. Finally, levels of glycosylation products were associated with a risk of clinical worsening, including hospitalization, lung transplantation, and all-cause mortality [61]. There is also strong evidence that HDL can improve glucose metabolism and insulin sensitivity in skeletal muscles and adipocytes. Low levels of HDL can predict development of type 2 diabetes mellitus and some medications used to raise HDL stimulate glucose metabolism and prevent diabetes mellitus [62,63]. Infusion of reconstituted HDL in patients with type 2 diabetes mellitus increased glucose disposal via mechanisms including increasing plasma insulin and activating AMP-activated protein kinase in skeletal muscle [63]. The relationship between HDL and glucose homeostasis is mediated by several different mechanisms, including HDL’s ability to stimulate insulin secretion by pancreatic beta cells, improving insulin resistance, and regulating lipid metabolism [40].

## 7. HDL and Platelet Activation

Platelet activation is another phenomenon associated with numerous mechanisms observed in the development of pulmonary vascular disease. Thrombotic lesions have been indicated as common pathological findings in patients with PAH [64]. Arteriopathy of the small pulmonary vessels with prominent thromboembolic changes was found in previous autopsy studies in PAH patients and was considered an important pathogenetic factor in this disease [65]. In physiological conditions, endothelial cells inhibit platelet aggregation by releasing nitric oxide and prostacyclin. This mitigates thrombosis and promotes fibrinolytic cascade by stimulation of the tissue plasminogen activator. In the case of pulmonary vascular injury, the platelet aggregation is accompanied by the release of vasoconstrictive and proinflammatiory factors. What is more, platelet’s granules themselves are a source of vasoactive molecules such as serotonin and tromboxane, growth factors, and proinflammatory cytokines such as soluble tumor necrosis factor-like weak inducer of apoptosis (sTWEAK), IL-1 β, transforming growth factor β, and TNF-α [65], all of which are proposed to play a role in the development of PAH. HDL exerts multiple anti-thrombotic properties by modulating increased platelet activity in patients with metabolic syndrome and diabetes. This platelet hyperreactivity, resulting in increased expression of surface receptors and adhesion molecules, enhanced production of thrombin and thromboxane A2, and dysregulated platelet calcium homeostasis, was reversed in vitro and in patients infused with reconstituted HDL. This effect was mainly attributed to reduction of cholesterol content of platelet membranes and lipid raft disruption [66]. Antiplatelet activity of HDL also includes interaction with platelet SR-BI, which in consequence leads to activation of protein kinase C, inhibition of calcium release, and upregulation of NO cellular production [67]. Finally, HDL is able to modulate pathways associated with PAH not only by stimulating NO synthesis but also by inhibiting platelet aggregation and plasma coagulation factors [66,68].

## 8. HDL and microRNA

**Figure 1:**
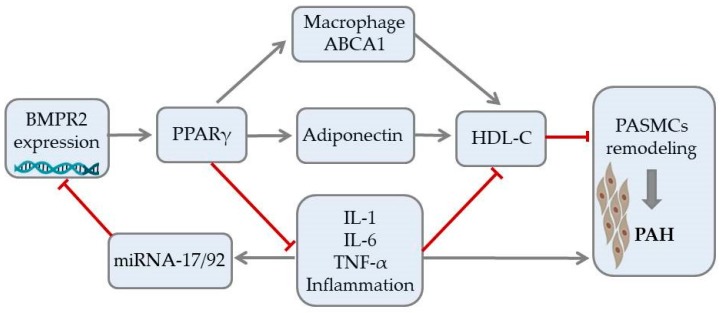
Potential signaling pathway between expression of bone morphogenetic protein receptor type II, HDL cholesterol, and vascular remodeling in pulmonary arterial hypertension. Arrows represent stimulation, T-bars represent inhibitory effect. ABCA1—ATP-binding cassette transporter 1, BMPR2—bone morphogenetic protein receptor type II, IL—interleukin, miRNA—microRNA, PAH—pulmonary arterial hypertension, PASMCs—pulmonary artery smooth muscle cells, PPAR-γ—peroxisome proliferator-activated receptor gamma, TNFα—tumor necrosis factor alpha.

Recent studies indicate that HDL particles can regulate gene expression by binding and transporting endogenous microRNA (miRNAs). miRNAs are involved in multiple cellular processes, including cell differentiation, proliferation, and apoptosis. An emerging body of evidence indicates a substantial role of miRNA signaling in maintaining homeostasis of the pulmonary circulation. Altered signaling of miRNA has been associated with the development of numerous PAH vascular hallmarks. These include endothelial dysfunction, smooth muscle cells proliferation, activation of fibroblasts, and formation of plexiform lesions [69]. The bone morphogenetic protein receptor type II (BMPR2) signaling plays an essential role in pulmonary circulation and BMPR2 mutations are the most common genetic cause of PAH [70,71,72]. Potential interaction between BMPR2 expression and HDL in PAH is shown in Figure 1. While dysfunction in BMPR2 signaling is unequivocally associated with pathogenesis of PAH, the exact molecular mechanisms involved in this pathway are still under investigation [73]. The latest reports revealed involvement of miRNA-BMPR2 axis in PAH pathogenesis. Activation of signal transducer and activator of transcription 3 by IL-6 was shown to induce the expression of the miR-17-92 cluster in pulmonary artery endothelial cells. Interestingly, miR-17-5p and miR-20a encoded in this cluster can target BMPR2 and, in consequence, downregulate expression of its protein [69]. It is, however, important to underline that cell-to-cell communication mediated by miRNA may be difficult because circulating miRNAs are quickly degraded. For that reason, most signaling miRNAs are bound to protective proteins or microvesicles, such as exosomes [74]. Interestingly, recent biophysical studies confirmed that extracellular miRNAs can bind to HDL through divalent cation bridging and thus remain shielded from external RNases. Due to its high radius of curvature and lower surface tension than other lipoproteins, HDL can facilitate binding a distinct pattern of miRNAs, which was recently described by Vickers et al. [75]. The HDL-miRNA profile measured by the amplification-based microarray platform was distinctly different from the exosome-derived profile. One of the most abundant miRNAs transported by HDL, miR-223, was also shown to be downregulated in PASMCs of PAH patients and has been associated with DNA damage in pulmonary artery smooth muscle cells. Restoration of lung miR-223 signaling was able to reverse vascular remodeling typical for PAH [76,77]. Until now, many clinical and experimental studies confirmed the association between circulating miRNAs and disease progression in PAH. The exact role of the HDL-mediated miRNA delivery pathway requires further research, but its discovery opened the discussion on potential miRNA-associated therapeutic targets in PAH [69,76,78]. 

## 9. HDL and Pulmonary Vascular Disease Severity 

The vasoprotective properties of HDL have been recently shown to play a significant role in patients with different forms of PAH. Heresi et al. showed that PAH subjects are characterized with lower HDL level than controls after adjusting for demographic factors and cardiovascular medical history. Patients with higher HDL were also characterized by longer time to clinical worsening than subjects with lower HDL values. What is more, in PAH patients, serum HDL concentration was able to differentiate between survivors and non-survivors [79]. In another paper, Zhao et al. divided 76 idiopathic PAH patients into groups with high and low HDL levels. The subgroup did not differ in terms of age, sex, or body mass index. Low HDL was associated with shorter six-minute walk distance, lower cardiac index, lower mixed venous oxygen saturation, and higher pulmonary vascular resistance. It was also established that serum HDL was an independent predictor of event-free survival in the two-year follow-up [80]. These findings were, however, questioned by a report from the French multicenter study of 110 PAH cases. The authors noted higher levels of HDL in PAH patients than in previously mentioned reports and did not find an association between HDL and three-year mortality [81]. Interestingly, in another large study by Larsen et al., in 227 PAH patients observed for over five years it was confirmed that higher HDL levels were associated with significantly lower mortality after adjusting for age, PAH therapy, and calculated disease-specific risk score [82]. To explain the relationship between serum HDL levels and clinical outcomes in PAH patients, the investigators from the Imperial College London analyzed metabolomics of two cohorts of patients with idiopathic and heritable PAH. They explored lipoprotein profiles of 204 subjects by nuclear magnetic resonance spectroscopy. They showed that downregulation of the smallest subclass of HDL (termed HDL-4) is associated with increased risk of mortality. Interestingly, HDL-4 particles can transport proteins such as prekallikrein, coagulation factor XI, and alpha-2-antiplasmin responsible for regulation of fibrinolysis, a mechanism likely to contribute to the observed clinical outcomes [83,84]. Explaining the mechanism in which lipoproteins can impact prognosis in debilitating and incurable disease seems promising in the context of novel therapies. In our recent study of 66 idiopathic PAH patients, we showed that HDL was able to predict pulmonary artery vasoreactivity and long-term response to calcium channel blocker therapy in patients with newly diagnosed disease. Additionally, we demonstrated that lower levels of HDL-C are correlated with inflammatory cytokines; this indicates that chronic inflammation can mediate the relationship between HDL-C and function of pulmonary vasculature [59,85]. The vasoprotective role of HDL was also recently described in 90 patients with chronic thromboembolic pulmonary hypertension (CTEPH). When compared to controls, CTEPH patients were characterized by lower HDL cholesterol levels but higher when compared to the PAH population. In this group higher HDL was associated with less prominent right ventricular dilation and a more favorable effect of pulmonary thromboendarterectomy [86]. This suggests that HDL cholesterol may be a useful marker in pulmonary vascular diseases of different origin.

## 10. Summary

Recent experimental and clinical data indicate protective properties of HDL in PAH, translating into survival benefits in long-term observations. However, the exact mechanism in which this lipoprotein fraction exerts its effect in pulmonary circulation is still under investigation and needs to be fully explored before it can be translated to novel diagnostic and treatment methods [87,88]. 

## Figures and Tables

**Table 1:** Classification and characteristics of different types of pulmonary hypertension.

Type of Pulmonary Hypertension	Pathogenesis	Hemodynamic Characteristics	Specific Drug Therapy
Pulmonary arterial hypertension Idiopathic Heritable Drugs and toxins induced Associated with Connective tissue disease HIV infection Portal hypertension Congenital heart disease Schistosomiasis Pulmonary veno-occlusive disease and/or pulmonary capillary haemangiomatosis Persistent pulmonary hypertension of the newborn	Usually multifactorial including alterations in: Genetics Inflammation Immunity Cell metabolism Hemodynamics	PAPm ≥ 25 mmHg and PAWP ≤ 15 mmHg and PVR > 3 WU	Calcium channel blockers Endothelin receptor antagonists Phosphodiesterase type 5 inhibitors Guanylate cyclase stimulators Prostacyclin analogues Prostacyclin receptor agonists
Pulmonary hypertension due to left heart disease	Passive backward transmission of filling pressures from the left heart	PAPm ≥ 25 mmHg and PAWP > 15 mmHg	Global management of the underlying condition of the left heart
Pulmonary hypertension due to lung diseases and/or hypoxia	Alveolar hypoventilation, vascular remodeling, parenchymal destruction, and fibrosis	PAPm ≥ 25 mmHg	Treatment of the underlying lung disease, long-term oxygen therapy in hypoxemic patients
Chronic thromboembolic pulmonary hypertension	Obstructive pulmonary artery remodeling as a consequence of vessel thromboembolism	PAPm ≥ 25 mmHg and PAWP ≤ 15 mmHg	Pulmonary endarterectomy, balloon pulmonary angioplasty, targeted medical therapy
Pulmonary hypertension with unclear and/or multifactorial mechanisms	Mechanisms are multifactorial and usually poorly understood	PAPm ≥ 25 mmHg	Treatment is tailored for underlying diagnosis; treatment of pulmonary hypertension is secondary

PAPm—mean pulmonary artery pressure, PAWP—pulmonary artery wedge pressure, PVR—pulmonary vascular resistance, WU—Wood unit.

**Table 2:** Potential mechanisms of vascular protection in pulmonary circulation.

Biological Activity of HDL	Potential Mechanisms of Vascular Protection
Vasodilatory activity	Stimulation of NO production Prostacyclin release Decreased production of reactive oxygen species
Anti-inflammatory properties	Downregulation of adhesion molecule Inhibition of monocyte activation Downregulation of proinflammatory macrophages Inhibition of NFkB and TNF-alpha signaling in endothelial cells
Antioxidative properties	Protection of LDL from oxidation Inhibition of cellular superoxide production
Cytoprotection	Protection of endothelial cells from apoptosis Modulation of mitochondrial electron transport Reduced cellular superoxide production Antiapoptotic signaling via ABCG1 Protection from extracellular matrix degradation by serpin peptidase inhibitors
Modulation of glucose metabolism	Stimulation of pancreatic insulin secretion Decrease of insulin resistance Modulation of cholesterol homeostasis Modulation of adipocyte metabolism
Regulation of platelet activation	Inhibition of platelet aggregation Anti-thrombotic effects on endothelium Upregulation of NO production Inhibition of calcium release
Regulation of gene expression	Transport of small non-coding microRNA

ABCG1—ATP-binding cassette sub-family G member 1, NFkB—nuclear factor kappa-light-chain-enhancer of activated B cells, NO—nitric oxide, TNF-α—tumor necrosis factor α.
